# Unusual Distribution of Cerebral Venous Thrombosis in a Patient With Sickle Cell Disease: A Case Study

**DOI:** 10.7759/cureus.48828

**Published:** 2023-11-15

**Authors:** Mohamed F Alchalban, Ahmed A Alekri, Shaikha M Alkaabi, Ali J Alhilly, Bedoor S Alomran

**Affiliations:** 1 Radiology, Bahrain Defence Force Royal Medical Services, Military Hospital, Riffa, BHR; 2 Internal Medicine, Salmaniya Medical Complex, Manama, BHR; 3 Internal Medicine, Bahrain Defence Force Royal Medical Services, Military Hospital, Riffa, BHR; 4 Neurology, Bahrain Defence Force Royal Medical Services, Military Hospital, Riffa, BHR

**Keywords:** cerebral venous thrombosis (cvt), sickle cell disease (scd), vaso occlusive crisis, internal cerebral vein thrombosis, sickle cell disease complications

## Abstract

This study presents the case of a 29-year-old Bahraini woman with a known history of sickle cell disease who exhibited acute neurological symptoms. Advanced imaging, specifically CT and MRI, identified cerebral venous thrombosis (CVT). The patient was managed with fluid therapy and anticoagulation, and received a packed red blood cell transfusion, leading to a complete recovery. Notably, this case was marked by the patient's positive anti-double stranded DNA (anti-dsDNA) status, typically linked with systemic lupus erythematosus (SLE), adding a potential pro-coagulant factor. The occlusion pattern, particularly involving the internal cerebral veins, was unique compared to other reviewed CVT cases in patients with sickle cell disease. This case emphasizes the significance of early diagnosis and intervention in CVT, especially in patients with sickle cell disease and other predisposing factors.

## Introduction

Sickle cell disease is an autosomal recessive inherited anemia. This disease arises from a genetic mutation that alters hemoglobin, the molecule responsible for oxygen transportation in the blood. Mutated hemoglobin becomes rigid and misshapen, impeding blood flow and leading to symptoms ranging from benign painful crises to severe ischemic events like stroke and acute chest syndrome [1]. While silent cerebral infarcts and stroke, both arterial in origin, are the most common permanent neurological complications in sickle cell disease [2], venous complications like cerebral venous thrombosis (CVT) can also emerge. CVT results when a thrombus forms in the large veins surrounding the brain, increasing intracranial pressure and potentially leading to ischemic or hemorrhagic stroke [3-4].

## Case presentation

A 29-year-old woman with a known diagnosis of sickle cell disease was admitted to the neurology department in the evening. She had presented with an acute onset of speech loss and abnormal behavior since the morning of the same day. As per her family's account, she complained of generalized body pain, predominantly in her right leg, accompanied by an unsteady gait. Notably, there was no report of chest or abdominal pain, urinary or gastrointestinal symptoms, or neurological complaints like syncope or facial asymmetry.

The patient has a history of regular vaso-occlusive crises throughout the year, averaging three to four hospital admissions annually, primarily due to vaso-occlusive and hemolytic crises. Despite her recurrent admissions, she has never been to the ICU and has no history of acute chest syndrome or stroke. Furthermore, she has never undergone an exchange transfusion, though she did require multiple simple top-up transfusions. Since 2015, she has been on daily hydroxyurea medication. Her last hospitalization, due to a crisis, was seven months prior. As for her social history, the patient is not married, has no children, and is not on any form of contraception. She does not smoke or consume alcohol, and there was no recent history of long-haul flight or surgical procedure.

On evaluation, vital signs were stable: oral temperature of 36.6°C, blood pressure of 132/68 mmHg, pulse rate of 109 bpm, respiratory rate of 16 breaths per minute, and oxygen saturation of 99% on room air. Neurologically, while she was alert, conscious, and oriented, she exhibited aphasia and was not responsive to questions. A comprehensive examination, including chest, cardiovascular, abdominal, and further neurological assessments, found generalized weakness but was otherwise unremarkable. Given her clinical presentation and history, the initial diagnosis pointed towards an ischemic stroke induced by her vaso-occlusive crises. However, other differentials were thought of as well, like hemorrhagic stroke or meningitis.

Upon admission, following a thorough initial clinical assessment, a comprehensive diagnostic workup was conducted. This included routine and specialized blood tests covering complete blood count, biochemistry, renal and liver function, cardiac biomarkers, coagulation screen, hemoglobin electrophoresis, and glucose-6-phosphate dehydrogenase (G6PD) deficiency assay (Table 1). Key findings included macrocytic anemia, a hemoglobin level of 8.6 g/dL, mild leukocytosis, and a positive urine culture for *Escherichia coli (E. coli)* (Table 2). Hormonal assays and specific pro-coagulant and rheumatological assays were within the normal range, with a notable exception of a positive double-stranded DNA (dsDNA) using enzyme-linked immunosorbent assay (ELISA), which can be associated with autoimmune conditions like systemic lupus erythematosus (SLE).

**Table 1:** Labs on admission aPTT, activated partial thromboplastin time; ANA, antinuclear antibody; AST, aspartate aminotransferase; CENP-B Abs, centromere protein B antibodies; OH, hydroxy; dsDNA Abs, double-stranded DNA antibodies; ESR, erythrocyte sedimentation rate; G6PD, glucose-6-phosphate dehydrogenase; MB, myocardial band; Hb, hemoglobin; HbA, hemoglobin A; HbA2, hemoglobin A2; HbC, hemoglobin C; HbF, hemoglobin F; HbS, hemoglobin S; INR, international normalized ratio; Jo-1 Abs, histidyl-tRNA synthetase antibodies; MCV, mean corpuscular volume; PLT, platelets; PT, prothrombin time; RETIC %, reticulocyte percentage; RNP 70, ribonucleoprotein 70 antibodies; Scl-70 Abs, topoisomerase I antibodies; Sm Abs, Smith antibodies; SS-A/Ro Abs, Sjögren's-syndrome-related antigen A antibodies; SS-B/La Abs, Sjögren's-syndrome-related antigen B antibodies; TSH, thyroid-stimulating hormone.

Variables	Values	Units	Reference ranges
Complete blood count			
WBC	11.87	x10^9^/L	4.00-11.00
RBC	2.18	10^12^/L	3.8-4.8
Hb	86	g/L	120-160
MCV	111.9	fL	78-100
PLT	397	x10^9^/L	150-450
RETIC %	6.25	%	0.5-2.50
Hematology			
ESR	29	mm/hr	0-15
Coagulation profile			
PT	15	s	13-15
INR	1.12		0.98-1.13
aPTT	28.1	s	28-45
D-Dimer	8.61	µg/mL	0-0.5
Electrolytes			
Sodium	136.9	mmol/L	136-145
Potassium	3.87	mmol/L	3.5-5.1
Chloride	104.8	mmol/L	98-107
Carbon dioxide	19.4	mmol/L	22-29
Renal function tests			
Urea	2.68	mmol/L	2.76-8.07
Uric acid	189	µmol/L	142.8-339.2
Creatinine	50.1	µmol/L	44-80
Calcium	2.48	mmol/L	2.15-2.5
Inorganic phosphate	1.14	mmol/L	0.81-1.45
Magnesium	0.87	mmol/L	0.66-1.07
Liver functions tests			
Total protein	77.4	g/L	64-83
Albumin	46.7	g/L	35-52
Globulin	30.7	g/L	23-35
Bilirubin total	12.1	µmol/L	0-24
Bilirubin direct	4.41	µmol/L	0-5
Bilirubin Indirect	7.69	µmol/L	3.4-12
Alkaline phosphatase	47.9	IU/L	35-104
Alanine aminotransferase	13.7	IU/L	0-33
G-glutamil transferase	19	IU/L	5-36
Cardiac markers			
AST	16.7	IU/L	0-32
Lactate dehydrogenase	214	IU/L	135-214
Creatine kinase	35.7	IU/L	20-180
Creatine kinase (MB)	11.6	IU/L	0-25
Troponin-I	0	µg/L	0-0.3
Hb electrophoresis			
HbA	0	%	95-98
HbA2	1.6	%	2-3
HbF	26.9	%	0.8-2
HbS	67.8	%	0
Acetyl HbF	3.7	%	0
HbC	0	%	0
G6PD test			
G6PD-quantitative	18.025	U/gHb	5.1-17
Special and hormonal assays			
TSH	2.62	µIU/mL	0.27-4.2
Free T4	19.1	pmol/L	12-22
Free T3	5.37	pmol/L	3.1-6.8
Thyroglobulin antibodies	13.6	IU/mL	0-115
Thyroid peroxidase antibodies	9	IU/mL	0-34
Vitamin B-12	118	pg/mL	211-946
Folate	31.3	ng/mL	5.3-19.3
Vitamin D (25-OH)	24.4	nmol/L	30-200
Molecular genetics			
Prothrombin c.20210G>A mutation	Negative		
Factor V Leiden c.1691G>A mutation	Negative		
Serology			
Beta-2-glycoprotein-1 abs. (IgG)	2	RU/mL	< 20
Beta-2-glycoprotein-1 abs. (IgM)	7	RU/mL	< 20
Anti-cardiolipin IgG	1.3	GPL-U/mL	10
Anti-cardiolipin IgM	3.4	MPL-U/mL	7
ANA screen	0.9		1-1.2
dsDNA Abs	21	IU/mL	20
Sm Abs	< 0.7	U/mL	15-25
SS-A/Ro Abs	< 0.3	U/mL	15-25
SS-B/La Abs	< 0.3	U/mL	15-25
CENP-B Abs	< 0.4	U/mL	10
Scl-70 Abs	< 0.6	U/mL	25
Jo-1 Abs	< 0.3	U/mL	25
Anti-RNP 70	< 0.3	U/mL	25

**Table 2:** Urine culture CFU, colony-forming unit; *E. coli, Escherichia coli*.

Bacteria	Results
E. coli	+3 (>100,000 CFU/ml)
Antibiotic resistance	
Ampicillin	Intermediate
Trimethoprim/sulfamethoxazole	Sensitive
Cefuroxime	Sensitive
Amoxicillin clavulanate	Sensitive
Levofloxacin	Sensitive
Nitrofurantoin	Sensitive
Ceftxime	Sensitive
Ciprofloxacin	Sensitive
Poperacillin/azobactam	Sensitive
Ceftazidime	Sensitive
Ceftriaxone	Sensitive

**Figure 1:**
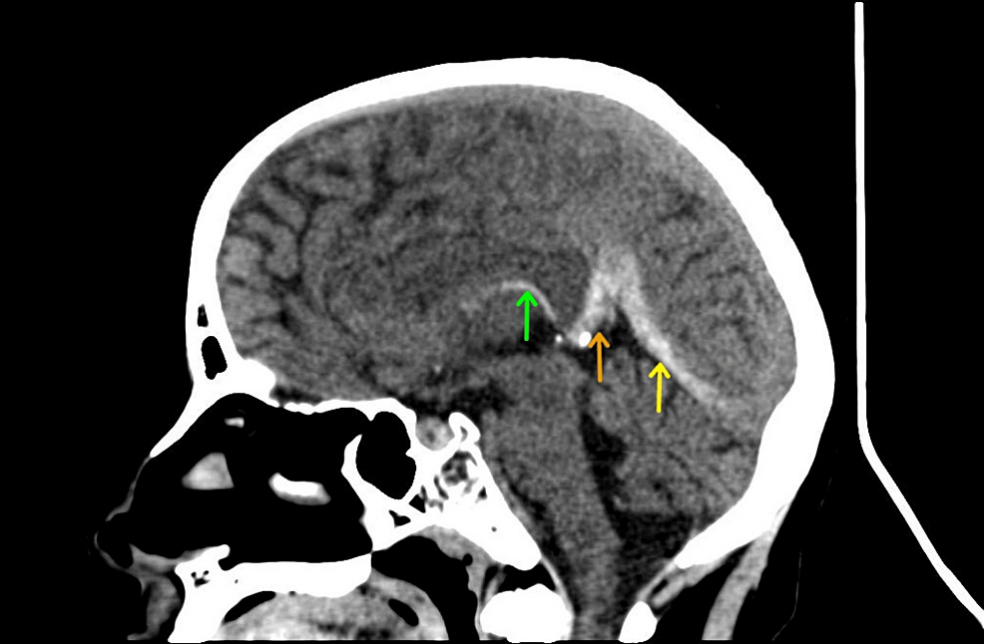
Sagittal non-contrast CT of the brain pre-treatment showing hyperdense internal cerebral vein (green arrow), the vein of Galen (orange arrow), and straight sinus (yellow arrow) consistent with cerebral venous thrombosis.

**Figure 2:**
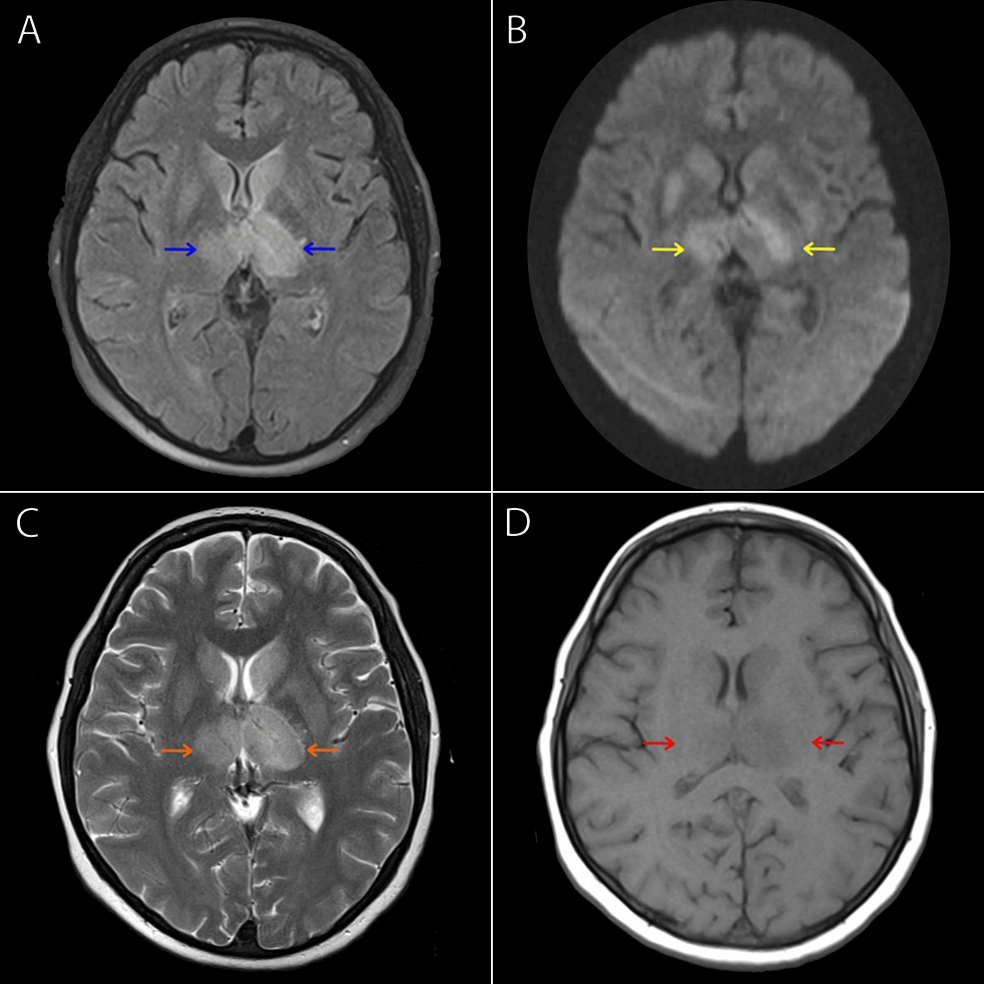
Multi-sequence MRI of the brain showing thalamus depicted in (A) T2 dark fluid sequence (blue arrows), (B) FLAIR sequence (yellow arrows), (C) T2 sequence (orange arrows), and (D) T1 sequence (red arrows). FLAIR, fluid-attenuated inversion recovery.

Further investigations with a CT scan of the head revealed thrombosis, particularly in the straight sinus, the vein of Galen, and the internal cerebral vein, with bilateral edematous thalami (Figure 1). A subsequent MRI confirmed the findings found on the CT imaging with possible partial involvement of the right transverse sinus, the right temporal region, and bilateral venous infarctions in the basal ganglia (Figures 2A-2D). A transesophageal echocardiogram was done which was non-significant with an ejection fraction of 60%.

The patient was diagnosed with CVT associated with sickle cell disease and bilateral thalamic infarcts, and she was managed with low molecular weight heparin, folic acid, fluid hydration, and a packed RBC transfusion.

**Figure 3:**
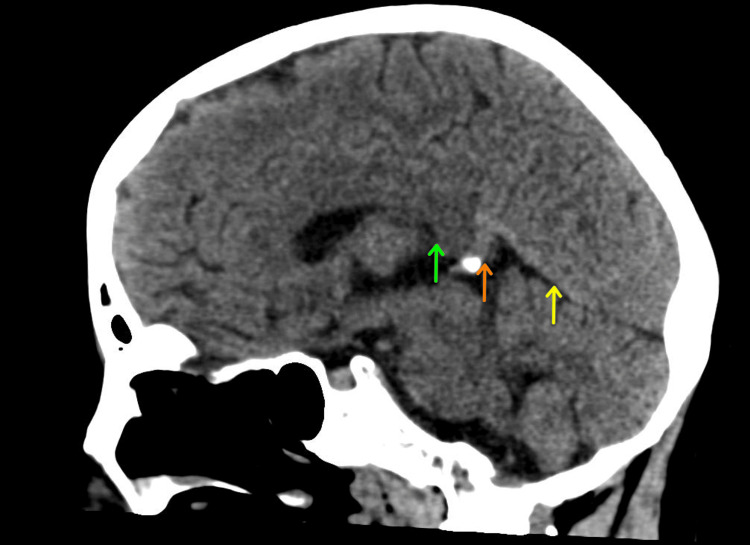
Sagittal non-contrast CT of the brain post-treatment showing complete resolution of the previously observed thrombosis in the internal cerebral veins (green arrow), the vein of Galen (orange arrow), and straight sinus (yellow arrow).

Prior to discharge, the patient improved significantly, she regained her full ability to speak and mobilize freely without help. A repeat complete blood count was conducted, revealing a normalization of the white cell count, an improvement in hemoglobin levels to 9.8 g/dL, and a normalization of the mean corpuscular volume (Table 3). Additionally, the reticulocyte count decreased to 2.5%. A subsequent CT scan showed reduced densities within the straight sinus and a reduction in edema in both the right temporal region and bilateral basal ganglia. After a seven-day hospital stay, the patient demonstrated significant improvement and was subsequently discharged with prescriptions for warfarin and folic acid. On follow-up visits, the patient continued to improve and no new symptoms were reported. A follow-up CT scan was scheduled three months post-discharge. This scan revealed no indications of acute cerebrovascular events (Figure 3). 

**Table 3:** Labs on discharge aPTT, activated partial thromboplastin time; Hb, hemoglobin; INR, international normalized ratio; MCV, mean corpuscular volume; PLT, platelets; PT, prothrombin time; RETIC %, reticulocyte percentage.

Variables	Values	Units	Reference ranges
Complete blood count			
WBC	6.98	x10^9^/L	4.00-11.00
RBC	2.8	10^12^/L	3.8-4.8
Hb	98	g/L	120-160
MCV	98.6	fL	78-100
PLT	515	x10^9^/L	150-450
RETIC %	2.53	%	0.5-2.50
Coagulation profile			
PT	14.5	s	13-15
INR	1.08		0.98-1.13
aPTT	31	s	28-45
Electrolytes			
Sodium	134.8	mmol/L	136-145
Potassium	4.1	mmol/L	3.5-5.1
Chloride	101.5	mmol/L	98-107
Carbon dioxide	21.5	mmol/L	22-29
Renal function tests			
Urea	2.79	mmol/L	2.76-8.07
Uric acid	139	µmol/L	142.8-339.2
Creatinine	36.1	µmol/L	44-80
Calcium	2.49	mmol/L	2.15-2.5
Inorganic phosphate	1.27	mmol/L	0.81-1.45
Magnesium	0.73	mmol/L	0.66-1.07
Liver functions tests			
Total protein	71.6	g/L	64-83
Albumin	43.1	g/L	35-52
Globulin	28.5	g/L	23-35
Bilirubin total	12.9	µmol/L	0-24
Bilirubin direct	4.75	µmol/L	0-5
Bilirubin indirect	8.15	µmol/L	3.4-12
Alkaline phosphatase	53.5	IU/L	35-104
Alanine aminotransferase	42.4	IU/L	0-33
G-glutamil transferase	20.6	IU/L	5-36
Cardiac markers			
Aspartate aminotrasnferase	33.8	IU/L	0-32

## Discussion

CVT is a critical, albeit rare, event that occurs when an obstruction, typically a blood clot, impedes the flow within the brain's venous sinuses, leading to blood accumulation and potentially culminating in a stroke [1]. While the incidence of CVT is approximately five per million individuals annually, certain groups, notably those with hemoglobinopathies such as sickle cell disease, are at an elevated risk [5-6].

A literature review uncovered 10 instances of CVT in patients diagnosed with sickle cell disease. Our patient, at 29 years of age, falls within the age range (18 to 37 years) identified in these cases; the average age reported was 26 years [7-15]. While the man-to-woman ratio was 7:3, the available data isn't sufficient to form definitive conclusions.

All the patients had a definitive history of sickle cell disease. The majority had frequent emergency visits due to painful vaso-occlusive crises. Only two reported a history of acute chest syndrome [7-14]. Our patient's presentation, characterized by generalized body pain, aphasia, ataxia, and confusion, aligns with a diagnosis of CVT and highlights cerebral involvement. Based on our review, seizures, including an incident of status epilepticus, were the predominant presenting symptom, occurring in four out of the 10 cases [8-10]. Other common neurological symptoms included photophobia, dysphasia, aphasia, and headache. Additional symptoms included hemiparesis, confusion, drowsiness, neck pain, facial weakness, facial pain, and chest pain [7-15].

Given the myriad of potential clinical manifestations, radiological imaging is indispensable in confirming a CVT diagnosis. Currently, CT and MRI scans are the most utilized imaging modalities for diagnosis [1]. For our case in particular, both imaging techniques were employed to reach the final diagnosis. A review of the literature revealed a slight preference for CT over MRI, potentially due to the similarity in presentation with stroke, where rapid hemorrhage exclusion is crucial [7-14]. Our patient's occlusion pattern was distinctive, involving the internal cerebral veins, the vein of Galen, and the straight sinus. This distribution was shared by only one of the other cases reviewed, albeit without the involvement of the internal cerebral veins [10]. Most other cases presented occlusions in either the sigmoid sinuses, transverse sinuses, or the superior/inferior sagittal sinus [7-9,11-13].

Therapeutic interventions varied across the cases. However, the foundational treatment approach focused on hydration, anticoagulation, and addressing accompanying symptoms or pathologies. A vast majority (nine out of 10) of the cases cited the use of anticoagulants [7-15]. Several other therapeutic strategies, ranging from antibiotics and antivirals to surgical interventions like craniotomies, were employed based on clinical indications [8,11-12,14].

Follow-up details were scant in the literature. However, available data indicated a generally positive prognosis [7-8]. In line with this, our patient's follow-up CT scan revealed no acute cerebrovascular complications.

A unique feature of our presented case lies in the observed anti-dsDNA positivity, commonly associated with SLE [6], thereby introducing an additional potential pro-coagulant factor. Further distinguishing our case was the occlusion pattern, particularly the thrombosis in the internal cerebral veins, a feature not found in the reviewed cases. Additionally, our case displayed a rare clinical presentation, with only one of the 10 other cases reviewed presenting with aphasia [15].

## Conclusions

While CVT remains a rare diagnosis, it is imperative to consider it in differential diagnosis, particularly in patients with non-specific symptoms and a history suggestive of blood disorders or coagulopathies. Fluid hydration therapy and anticoagulation are cornerstones of CVT management. Continued research and a more extensive body of published cases are essential to enhance our understanding of the pathophysiology of CVT, its management, and the progression of the disease.

The rarity of cerebral venous thrombosis in patients with sickle cell disease necessitates this case's documentation. It demonstrates that timely clinical and radiological assessment, combined with appropriate management, can lead to favorable outcomes. Our patient's complete recovery with conservative treatment underscores the importance of early diagnosis and intervention. A deeper exploration of the disease, especially in patients with additional predisposing factors, can provide better insights into its pathophysiology.
